# Comprehensive Review of Tinea Capitis in Adults: Epidemiology, Risk Factors, Clinical Presentations, and Management

**DOI:** 10.3390/jof10050357

**Published:** 2024-05-16

**Authors:** Rachel C. Hill, Jeremy A. W. Gold, Shari R. Lipner

**Affiliations:** 1 Weill Cornell Medical College, New York, NY 10065, USA; 2 Mycotic Diseases Branch, Centers for Disease Control and Prevention, Atlanta, GA 30329, USA; 3 Department of Dermatology, Weill Cornell Medicine, New York, NY 10021, USA

**Keywords:** tinea capitis, adult tinea capitis, trichophyton, microsporum, kerion, favus, ectothrix, endothrix, antifungal resistance

## Abstract

Tinea capitis is a fungal infection of the scalp and hair caused by dermatophyte molds, that most often affects children and may also affect adults. Previous estimates suggest that between 3% and 11% of all tinea capitis cases worldwide occur in adults, although updated epidemiological studies are needed to reassess the prevalence of tinea capitis in adult populations specifically. Postmenopausal adult women are most often affected by tinea capitis, with African American or Black women particularly at risk. Adults who experience crowded living conditions, who live in close proximity to animals, who are immunosuppressed, and/or who live in households with affected children are at greatest risk of infection. Tinea capitis can be non-inflammatory or inflammatory in nature, and the subtype affects the extent and severity of clinical symptoms. Fungal culture and potassium hydroxide preparations are the most commonly used diagnostic tools. Trichoscopy, defined as dermoscopic imaging of the scalp and hair, is a useful adjunct to the physical examination. The mainstay of therapy is oral antifungal therapy, and topical therapy alone is not recommended. Since tinea capitis infection is uncommon in adults, there are no widely accepted treatment guidelines. Rather, the same medications used for tinea capitis infection among children are recommended for adults at varying doses, including griseofulvin, and terbinafine, and, less commonly, itraconazole and fluconazole. The prognosis for tinea capitis in adults is typically excellent when prompt and adequate treatment is administered; however, delayed diagnosis or inadequate treatment can result in scarring alopecia. Over the past decade, dermatophyte infections resistant to treatment with topical and oral antifungal agents have emerged. While tinea capitis infections resistant to antifungal therapy have been rarely reported to date, antifungal resistance is rising among superficial fungal infections in general, and antifungal stewardship is necessary to ensure that resistance to treatment does not develop among dermatophytes that cause tinea capitis.

## 1. Introduction

Tinea capitis (TC) is a fungal infection of the scalp and hair caused by dermatophyte molds [1,2]. Although geographic distribution varies worldwide, the main causal organisms of TC are species in the *Trichophyton* and *Microsporum* genera, namely *Trichophyton tonsurans*, an anthropophilic species, and *Microsporum canis*, a zoophilic species [3]. TC is the most common dermatophyte infection in children, both in the United States (US) and worldwide, especially in children who are people of color, especially Black children and/or children of LatinX descent, and those between the ages of three and seven years [4,5,6]. The clinical manifestations of TC depend on the species of dermatophyte causing infection. The clinical findings associated with TC range from asymptomatic or mild pruritus and scaling to extensive inflammation and alopecia and depend on the extent of infection and host susceptibility [5]. Although less common than pediatric TC, physicians may neglect to consider TC infection as part of a broad differential diagnosis in adults, leading to delayed diagnosis and the possibility of poor outcomes, including permanent scarring. Consequently, an updated review on TC in adults is warranted. This clinical review is intended to provide a comprehensive update of TC infections in adults, including epidemiology, risk factors, clinical presentations, and management and treatment in older populations.

## 2. Geographic Distribution of Causative Organisms

In general, dermatophyte species that cause TC are either anthropophilic, spread by human-to-human transmission, or zoophilic, spread by animal-to-human transmission [7,8]. The two major species responsible for TC worldwide are *T. tonsurans* and *M. canis* [7]. TC infections in Europe and Asia are generally due to *M. canis*, while those in North and South America and the United Kingdom are generally due to *T. tonsurans* [7,9,10]. In parts of Africa, Asia, and Eastern Europe, certain rarer dermatophytes are endemic, including *Trichophyton violaceum*, *Trichophyton schoenleinii*, and *Microsporum audouinii* [7,11,12,13,14]. *Trichophyton soudanense* is also endemic across the African continent [15,16]. Due to climate change, global migration, and fervent antifungal use, the geographic distribution of dermatophyte species responsible for TC infection may change over time [7,8]. Diagnosing dermatophytes to the species level is a time-consuming process, typically requiring microscopic examination followed by culture. Molecular diagnostics, including polymerase chain reaction (PCR), can be used to identify species more rapidly, although these protocols are cost intensive [17]. Until PCR is widely adopted to identify causative species, the accuracy of species distribution estimates is likely to be limited. (See Section 10, Diagnostic Methods, for further discussion of PCR as a diagnostic method to identify causative species.)

## 3. Epidemiology of Tinea Capitis in Adults

Previous estimates have suggested that between 3% and 11% of all TC cases worldwide occur in adults [18,19,20,21]. However, these studies are now outdated, and more current, large-scale studies are needed to characterize the epidemiologic landscape of TC in adults specifically. Although TC infections occur worldwide, they are more common in developing countries with tropical or subtropical climates [22]. Furthermore, socioeconomic conditions, including overcrowding and living in close proximity to animals, may influence the prevalence of TC infections in certain communities across the world [22].

Adult women may be more frequently affected by TC than adult men. In a case series of an *M. canis* TC infection outbreak at a nursing home in Belgium by Hillary et al., all eight patients included were women over the age of 70 [23]. Similarly, in a case series of eight adult patients with TC at a single dermatology department in France, six out of eight patients were women [20]. Women in the postmenopausal years may be particularly susceptible, possibly due to decreased sebum production caused by a reduction in estrogen levels [8,20]. In a retrospective study of 269 potassium hydroxide (KOH) confirmed cases of adult TC in mainland China by Liang et al., 137 patients were between the ages of 45 and 89 years, and the sex ratio was 1:5.2 male to female [24]. However, data from low-income countries are lacking confirmation as to whether the sex distribution of TC infections is consistent on a global scale. Large, prospective studies are needed to verify female predominance in a wide range of geographic distributions.

In the US, TC infections are most prevalent in African American children. In a retrospective study of 672,373 children aged below 15 years in Northern California who received a diagnosis of TC or first-time antifungal prescription, African American children had higher incidence ratios of TC compared with White children (*p* < 0.001) [9]. In another retrospective study of TC among 4,148,385 children < 18 years old, who were Medicaid-insured, the highest incidence rate of TC was in non-Hispanic Black children, with incidence rates 6.7 times higher than in non-Hispanic White children [6]. Similarly, African American women may be at greater risk of developing TC infections. In a retrospective study of 79 cases of TC at a single urban institution in the US serving predominantly Hispanic or Latino and Black or African American persons, 9 cases involved adults, 7 of whom were African American women [19]. Of the remaining two cases, both occurred in African American men. Hypotheses for this disproportionate representation of TC in African American women include roles as primary caretakers for children who are affected by TC, and hair care practices, including less frequent shampooing and traction hair styling that may allow fungal pathogens to penetrate the hair and scalp more easily [19]. However, a case–control study of 66 patients aged 12 years and younger in three urban referral centers in the US found that hair care practices, including hairstyling, washing frequency, and use of oils, were not associated with the presence of TC in children (*p* > 0.05) [25]. Therefore, it remains unclear whether the increased TC risk observed in Black or African American women is due to decreased sebum production with age or increased sebum due to infrequent hair washing, or other factors. Future studies are needed to determine whether hair care practices among adult women play a role in the pathogenesis of adult TC.

## 4. Transmission

The transmission of TC may be direct or indirect. For direct transmission, persons or animals harboring TC spread the infection to an uninfected person via close contact [26]. For indirect transmission, uninfected persons encounter fomites via contact with hair or skin scales in their vicinity or via contact with infected belongings, such as clothing, blankets, towels, or tools used for hair grooming [26,27,28]. Asymptomatic carriers, in whom dermatophytes can be detected on the scalp without any clinical manifestations of infection, may also play a role in perpetuating transmission and in endemic TC infections worldwide. Consensus on management of asymptomatic carriers is currently lacking [5].

## 5. Risk Factors for Tinea Capitis in Adults

Several factors may make certain adults more susceptible to TC infections. Persons at the extremes of age, including young children and older adults, are at greater risk for TC infection. The onset of puberty provides protection from TC, due to increased sweat, sebum, and hair thickness [29]. Older, post-menopausal women, in particular, may be at increased risk of dermatophytosis due to decreased sebum production associated with decreased estrogen levels, resulting in decreased fatty acid production and loss of acidity on the scalp [8,30,31,32]. Older adults are more likely to live in group homes, including nursing homes or assisted living facilities, leading to greater opportunity for exposure to asymptomatic carriers and fomites [23,33].

While other dermatophyte infections became more prevalent due to increased global mobility following World War II, TC infections declined due to improvement in personal hygiene practices [7]. However, poor hygiene continues to play a role in the development of TC infections in the modern day. Circumstances in which people are in close contact with infected persons or belongings promote TC infection. Crowded living conditions, including group homes and army barracks, contact with infected children, contact sports, and using shared gym or swimming pool facilities are common situations in which persons may be exposed to dermatophyte infections [8,19,26,32,34,35,36,37]. In the case series of an *M. canis* TC infection outbreak involving six patients in Belgium by Hillary et al., the patients lived in close proximity inside of the same nursing home, highlighting the potential for human-to-human transmission among older adults [23].

Living in close proximity to animals also puts persons at risk for developing TC. Both domestic animals, including cats and dogs, and livestock, including cattle, may transmit TC infection to humans [38]. *M. canis* TC is particularly associated with transmission from cats and dogs that live as house pets [38]. Notably, the pets may not exhibit any signs of fur or skin disease. In the retrospective study of 269 cases of adult TC in mainland China by Liang et al., 52 patients (19.3%) reported a history of contact with animals, most often cattle, cats, and dogs [24].

TC is also commonly spread through contact with fomites, and proper sanitization of shared objects can help prevent the spread. A study by Akhoundi et al. explored the effect of laundering on the survival of dermatophyte conidia on contaminated gauze pads, including *T. tonsurans*, which were laundered at 40 °C, 60 °C, or 90 °C in domestic washing machines, and subsequently incubated at 27 °C for 3 weeks on a Sabouraud glucose agar plate and monitored for dermatophyte growth. The study showed that 60 °C laundering was sufficient for killing conidia on the infected pads [26,39]. Improper cleaning techniques, such as the use of cold water, when laundering blankets, pillows, textiles, or when sanitizing shared hair tools may increase the risk of TC infection. In a case series of 18 male patients aged 4–24 years old with culture or molecular confirmation of TC infection, all patients had reported receiving a haircut in a barber shop within 2 weeks preceding the onset of infection [28]. Shaving and haircutting may cause microtrauma, allowing access for the pathogens to penetrate the scalp and/or hair [28]. In a prospective study of 41 samples taken from fomites in hairdressing salons in Mali, West Africa, including needles used for braiding, hairbrushes, scissors, razors, and combs, dermatophytes were isolated from 30 samples (73.2%), suggesting that hair salons and barber shops may be a key infection source [27].

Immunosuppressed adults appear to be at greater risk for TC, particularly with *M. canis* [20,29,40,41,42]. Causes of immunosuppression associated with TC include malignancy, transplantation, human immunodeficiency virus (HIV) infection, diabetes mellitus, or corticosteroid therapy [20,29,40,41,42]. In a study of eight cases of mycologically confirmed adult TC in France, patient comorbidities included non-Hodgkin lymphoma (n = 2), thrombocytopenia (n = 1), systemic corticosteroid use (n = 1), and HIV positivity (n = 1), with four of the eight being infected with *M. canis* [20]. In a retrospective study including 121 mycologically confirmed TC cases at a dermatology clinic in Tehran, Iran, 25 patients were adults. Of these, 80% (n = 20) were immunocompromised, due to diabetes mellitus, frequent topical or oral corticosteroid use, HIV infection, and transplantation [8]. Fourteen of the twenty immunocompromised patients with TC had fungal co-infection of the foot, trunk, nail, hand, and/or groin [8]. In a multi-center study of 1380 patients, including 58 adults, diagnosed with TC at academic centers in Egypt, 34.4% (n = 20) of patients were co-infected with hepatitis C, of which 45% were undergoing interferon alpha therapy (n = 9), and 22.4% (n = 13) were diagnosed with diabetes mellitus [31]. While immunosuppressed patients are likely at increased risk of TC, adults with no apparent risk factors can also contract TC. In the retrospective study of 269 cases of adult TC in mainland China by Liang et al., only 6 cases occurred in patients with a reported immunocompromised state (2.2%) [24].

Concurrent fungal infection of another anatomic area is associated with TC via autoinoculation [30]. In a retrospective study of 82 adults with mycologically confirmed TC infection at a tertiary hospital in Korea, 48 patients were diagnosed with an additional cutaneous fungal infection, most commonly tinea faciei (n = 32), tinea corporis (n = 8), and tinea pedis (n = 3) [43]. Therefore, it is important to both screen for TC in patients diagnosed with cutaneous fungal infections involving sites besides the hair and scalp, and examine patients for the presence of cutaneous fungal infections at other anatomic sites when TC is diagnosed.

## 6. Classification

TC can be divided into noninflammatory and inflammatory subtypes, which generally predicts the clinical presentation. The noninflammatory subtype comprises both ectothrix and endothrix infections, which represent two distinct types of hair shaft invasion [44]. In ectothrix infections, arthroconidia invade at the level of the mid hair shaft and adhere to its exterior. Hyphae then grow toward the bulb of the hair [44,45,46]. Ectothrix infections typically fluoresce under Wood’s light and are mainly caused by *Microsporum* species or *Trichophyton verrucosum* [46]. In endothrix infections, arthroconidia invade the hair shaft itself. Endothrix infections generally do not fluoresce under Wood’s light, and are typically caused by *T. tonsurans*, *T. soudanense*, or *T. violaceum*.

For the inflammatory subtype, it is hypothesized that there is a hypersensitivity reaction to the dermatophyte infection and includes favus and kerion [2]. Favus, the third type of hair shaft invasion, is characterized by the development of long hyphae and air spaces within the hair shaft that can be appreciated with KOH microscopy [47]. Favus is most frequently caused by the anthropophilic species *T. schoenleinii* [46,47]. Kerion is caused by a delayed immune response to dermatophyte infection and is typically caused by zoophilic species [48].

## 7. Clinical Presentation and Physical Exam Findings

**Figure 1:**
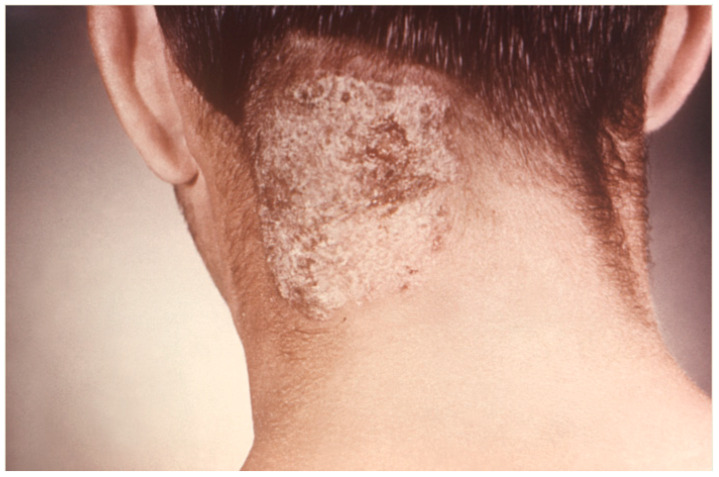
Posterior neck and scalp tinea capitis caused by unknown dermatophyte (Centers for Disease Control and Prevention).

**Figure 2:**
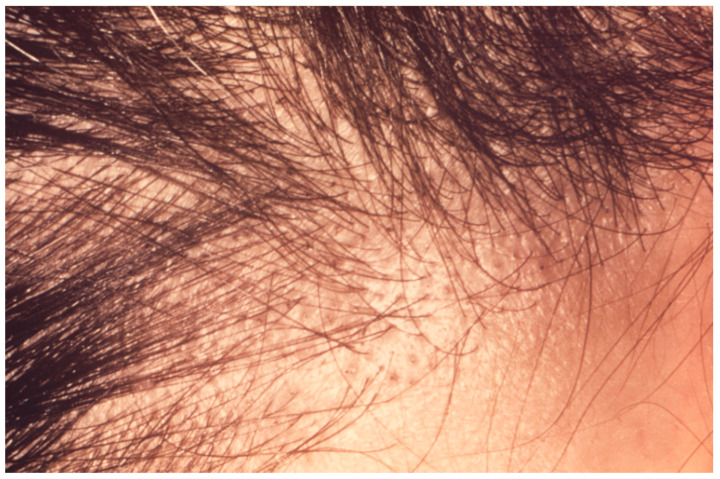
Scalp of an adult male patient with alopecia secondary to *T. violaceum* TC (Centers for Disease Control and Prevention/Dr. Lucille K. Georg).

**Figure 3:**
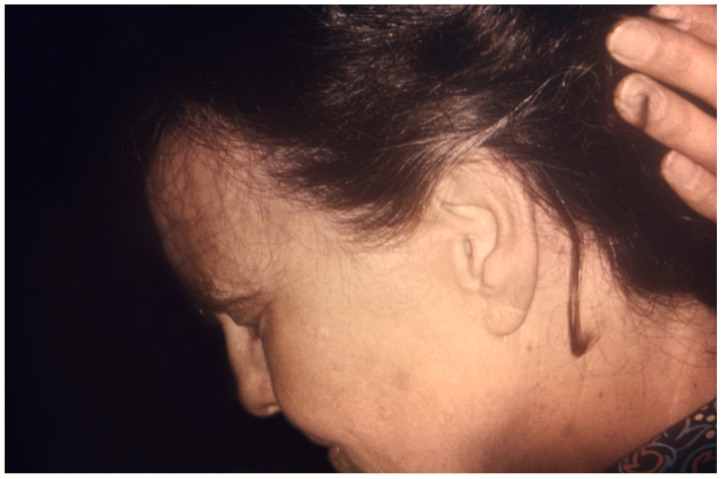
Adult female patient with *T. violaceum* tinea capitis and subtle hair loss (Centers for Disease Control and Prevention/Dr. Lucille K. Georg).

Non-inflammatory TC in adults is often accompanied by alopecia, for both ectothrix or endothrix infections (Figure 1, Figure 2 and Figure 3) [44]. In an 18-year retrospective single-center study of TC cases in 82 adults in Korea by Park et al., 46 patients (56.1%) presented with hair loss [43]. Ectothrix infections may appear on the scalp as single or multiple scaly, erythematous patches with mild inflammation accompanied by circular areas of hair loss that may appear grey, known as “grey-patch ringworm”. Ectothrix-type TC can also appear as diffuse fine white scaling of the scalp with subtle hair loss that resembles seborrheic dermatitis, known as the seborrheic type [43,45,46]. In ectothrix TC, the hair shaft breaks 2–3 mm or more above the scalp due to the invasion pattern. In the retrospective study of TC cases in 82 adults in Korea by Park et al., *M. canis* infection was the most common causal organism, with 25.6% (n = 21) of patients presenting with seborrheic dermatitis-like scaling and 23.2% (n = 19) of patients presenting with grey patch TC [43].

Endothrix infections typically result in breakage of the hair at the level of the scalp, leaving stubs of the proximal hair shaft behind within the follicle and areas of well-demarcated hair loss [45]. This creates the appearance of black dots on a patch of bald scalp, leading to the common name “black dot ringworm” [49]. In a retrospective study of 111 adults with *T. tonsurans* tinea capitis confirmed by fungal culture or PCR, 78.9% of patients experienced the non-inflammatory endothrix form of infection and 87.6% of patients presented with occipital scalp involvement [37].

Inflammatory TC infections, including both favus and kerion, can lead to alopecia accompanied by permanent scarring and disfigurement, depending on the severity and extent of infection [47]. While uncommon in the United States and Western Europe, favus is still endemic in parts of Africa, and also found in the Middle and Far East of the Asian continent [50]. Favus has an insidious presentation and progresses in three stages: stage one involves erythema surrounding the hair follicles, and stage two involves the development of a scutulum, which forms within a hair follicle. Scutula are keratotic, yellow, crusted concave plaques on the scalp that can cause extensive hair loss. They contain hyphae and neutrophils and can increase susceptibility to secondary bacterial infection [47,48,51]. In stage three, the most severe stage, multiple scutula may coalesce, leading to extensive hair loss and possible scarring [47]. Due to its chronic nature, favus can persist into adulthood, even if acquired in childhood or adolescence, and fails to resolve with the onset of puberty [45,47]. In the retrospective review of 269 clinical cases of TC in China from 2000 to 2019 by Liang et al., only 4 patients (1.5%) had favus [24].

Kerion is the most severe presentation of inflammatory-type TC and is believed to be more common in rural populations due to increased contact with animals [52]. Kerion is characterized by a solitary, boggy, inflammatory plaque, usually on the occipital scalp, with purulent discharge from the hair follicles [46]. Patients typically present with pain in the affected area, fever, and lymphadenopathy [46]. While most patients have a confined area involved, a larger area of infection may result from multiple plaques that coalesce in rare circumstances [46,53]. John et al. proposed major and minor features to distinguish kerion and a grading system to classify the severity of infection. Major criteria include tenderness to palpation, alopecia, pustules and purulent drainage, and scaling, while minor criteria include dermatophytid reaction, defined as an immunological reaction caused by infection, regional lymphadenopathy, short hairs apparent on dermoscopy, boggy plaques, clear demarcation of borders, overlying erythema, and pruritus [53]. Severity is graded on a scale from one to four, with grade one defined as a few pustules overlying an erythematous plaque, while grade four is defined as pustules and erythema accompanied by alopecia and scarring [53]. In the retrospective review of 269 clinical cases of TC in China from 2000 to 2019 by Liang et al., 84 patients (31.2%) presented with kerion compared to 4 patients with favus (1.5%), suggesting that kerion is more prevalent than favus for inflammatory TC [24].

## 8. Trichoscopy

Trichoscopy, defined as dermoscopic imaging of the scalp and hair, is a useful tool to aid in the physical examination of suspected TC. Typical trichoscopic features of TC include short, broken hairs and scales, and, depending on the causative organism, may include black dots, corkscrew hairs, Morse code-like or barcode-like hairs, comma-shaped hairs, and zigzag hairs [54]. A systematic review of the trichoscophic findings of TC, including 37 articles, found that comma hairs, corkscrew hairs, Morse code-like hairs, and zigzag hairs had the highest predictive value for TC diagnosis (51%, 32%, 22% and 21% prevalence, respectively), with Morse code-like hairs (n = 8/29), zigzag hairs (n = 6/29), bent hairs (n = 4/29), and diffuse scaling (n = 4/29) only seen in TC infections secondary to *Microsporum* species (all *p* < 0.05), while corkscrew hairs (n = 21/38) were more commonly found in TC infections secondary to *Trichophyton* species (*p* < 0.001). Comma hairs, black dots, and broken hairs were equally likely to be found in *Microsporum* and *Trichophyton* TC infections (*p* = 0.42, *p* = 0.73, and *p* = 0.99, respectively) [55]. Because identification of the causative organism may guide treatment of TC, following confirmation of TC via KOH with microscopy, trichoscopic findings may be used to select empiric therapy while fungal culture results are pending, which can take up to a month [56]. Trichoscopy may also be used to rapidly assess treatment response once therapy is initiated [55,57].

## 9. Differential Diagnosis

The differential diagnosis of adult TC is broad, due to an array of other conditions that lead to scaling and erythema of the scalp with or without pustules and alopecia, and due to the many possible clinical presentations of TC depending on infection pattern and causal organism. We recommend that the skin and nails of patients presenting with scaling and erythema of the scalp are also carefully examined to glean a complete clinical picture of the level of TC infection [58,59]. For example, suspicion of TC infection may increase if a patient also presents with an erythematous, scaly, ring-shaped rash on the body that is typical of tinea corporis. Conversely, suspicion of TC infection may decrease if a patient also presents with erythematous plaques with silvery scale in lighter-skinned patients or violaceous to hyperpigmented plaque with greyish scale on the knees and elbows, or multiple nails with pitting and onycholysis, which would instead indicate psoriasis. Since lymphadenopathy may accompany TC, cervical lymph node palpation is recommended [46]. In the retrospective study of 185 cases of TC in Korea, including 82 adults, the most common differential diagnoses other than TC, included seborrheic dermatitis (n = 20, 24.4%), folliculitis (n = 15, 18.3%), allergic contact dermatitis (n = 5, 6.1%), and psoriasis (n = 4, 4.9%). Other less common initial diagnoses included dissecting cellulitis, eczema, telogen effluvium, lupus erythematosus, and acne [43]. Adult patients with these diagnoses who do not improve after treatment should be evaluated for possible TC infection with a mycological examination.

## 10. Diagnostic Methods

The first step in the diagnosis of TC in adult patients is a thorough physical examination coupled with trichoscopy (see Section 7, Clinical Presentation and Physical Exam Findings and Section 8, Trichoscopy, above). If there is clinical suspicion for TC infection, it is recommended that the diagnosis is confirmed using fungal culture before treatment initiation [58]. Although results may take anywhere from 2 to 8 weeks, fungal culture with Mycosel agar, or other enriched agars containing cycloheximide and chloramphenicol, is the gold standard for TC diagnosis [33]. Samples may be collected for fungal culture using a scalpel blade, hairbrush, toothbrush, cotton swab, or gauze, depending on the practice setting and availability of resources [33,60,61,62]. In cases in which the clinical presentation is highly indicative of kerion, false negative results do not rule out TC infection. Because a kerion is often accompanied by purulent discharge and crust, it is unlikely that the fungal organisms can be cultured from this area [63]. In such cases, collecting a sample of co-occurring, non-inflammatory TC lesions when possible is encouraged [63]. While fungal culture results are pending, 10–20% KOH preparation may be used to examine proximal hairs for evidence of TC infection [64]. The hair sample is applied to a glass slide, impregnated with KOH, and viewed under the microscope to evaluate for fungal spores inside or outside of the hair shaft. In a prospective study including 25 patients with TC, KOH wet mount and culture on Sabouraud’s dextrose agar were both positive in 17 patient samples (both 68%). The sensitivity, specificity, positive predictive value, negative predictive value, and accuracy for KOH wet mount were 80%, 88%, 90%, 82%, and 85%, respectively, vs. 92%, 86%, 88%, 90%, and 89%, respectively, for culture on Sabouraud’s dextrose agar (*p*-value not reported), suggesting that culture may be more sensitive, while KOH may be more specific, for identifying TC infection. However, future studies are needed to directly compare the two diagnostic methods to corroborate this finding [65].

Polymerase chain reaction (PCR) and reflectance confocal microscopy (RCM) are two newer, less commonly used, methods for diagnosing TC. Nested PCR is defined as sequential amplification reactions to reduce nonspecific amplification of the DNA template. In a prospective study evaluating the performance of nested PCR in detecting the chitin synthase 1 gene in 155 patients with suspected dermatophytosis, including 50 patients with hair involvement, nested PCR was most often positive (n = 43/50, 86%) vs. KOH microscopy (n = 29/50, 58%), first-round PCR (n = 26/50, 52%), and fungal culture (n = 15/50, 30%). In originally negative KOH microscopy (n = 21) and culture-negative (n = 35) specimen samples, nested PCR was positive in 66.6% and 80% of cases, respectively, when applied to these same samples [66]. Similarly, in a cross-sectional, descriptive, 3-year study of 129 hair samples from patients ranging in age from 1 to 80 years old (mean age 23.64) subjected to a commercial multiplex real-time PCR assay, the PCR assay was more sensitive compared to fungal culture (sensitivity 89.3%, specificity 75.3%, positive predictive value 73.3%, negative predictive value 90.2%, accuracy 81.4%, *p* < 0.05) [67]. Although PCR may be more reliable in identifying TC infections compared to KOH microscopy and fungal culture, PCR has not been widely adopted as the gold standard due to the complexity of the method and lack of resources and trained staff in smaller laboratories [66].

RCM is a non-invasive imaging technique that can be used either in vivo or ex vivo to detect dermatophytosis of the hair and/or scalp in minutes. In a study of hair dermatophytosis in six people, including two adult patients, conidia were visible in or on the affected hair shaft due to high reflectance properties [68]. In vivo RCM is useful for analyzing the scalp in conjunction with the hair, but ex vivo technique of extracted hair must be used if the patient presents with an inflammatory-type TC infection due to concerns of impaired image quality [68]. Despite its potential to quickly identify dermatophyte infections involving hair, RCM is not commonly performed due to the high cost of equipment and lack of training [68,69,70].

## 11. Treatment and Management

Similar to the treatment of TC in children, the mainstay of therapy for adult TC is oral antifungal therapy (Table 1). Topical antifungal therapy is typically not recommended due to poor penetration of the hair and scalp, rendering it ineffective [71,72]. Since TC infection is uncommon in adults, there are no widely accepted treatment guidelines. Rather, the same medications used for TC infection among children are recommended for adults at varying doses, including griseofulvin, and terbinafine, and less commonly, itraconazole and fluconazole [34,37,52,73,74,75,76,77,78,79,80]. Regardless of the selected therapy, it is recommended to re-evaluate patients approximately one month after treatment initiation to assess for improvement [72]. If there is no response to therapy, the choice of antifungal may be reconsidered, or the differential revisited to ensure that the correct diagnosis was made [72].

Griseofulvin is a United States (US) Food and Drug Administration (FDA) approved treatment for TC and is typically considered first-line treatment for TC among children and adults alike, especially in cases where the causative species is unknown or cannot be identified. However, large, randomized controlled trials evaluating the efficacy of griseofulvin vs. other systemic antifungal therapies in adults specifically are lacking. In a case series of three adult patients with culture-confirmed TC infection by Vidimos et al. (*T. tonsurans* n = 2, *M. canis* n = 1), all achieved clinical cure and experienced regrowth of hair in areas of alopecia after treatment with oral griseofulvin 250 mg twice daily for 2 to 4 months [73]. In a retrospective study of 121 cases of inflammatory-type TC caused by *Trichophyton interdigitale* complex, *T. violaceum*, and *M. canis*, across 12 years, including 6 adults with an average age of 31 years, treated with 1 g of griseofulvin per day for 6–8 weeks, clinical cure was achieved in 5 out of 6 cases. One woman, who had a relapsed gastric tumor, required > 3 months of griseofulvin treatment [52]. In a retrospective study of 60 adult patients ≥ 18 years old with mycologically confirmed TC (*T. violaceum* n = 36, *M. canis* n = 12, *T. schoenleini* n = 7, *T. verrucosum* n = 2, *Trichophyton mentagrophytes* n = 1, and *Trichophyton rubrum* n = 1) at a single center in Tunisia, in which all patients were treated with griseofulvin 20–25 mg/kg/day for 6 weeks (n = 48) or 8 weeks (n = 12) and topical antifungals once or twice daily, complete cure was achieved in 55 patients, while relapse requiring an additional course of griseofulvin occurred in 2 cases, with 2 patients lost to follow up [34]. The recommended dosage for griseofulvin is now 10–15 mg/kg/day for at least 8 weeks [74,75].

Terbinafine is also a US FDA-approved treatment for TC infection. In a retrospective cohort study of 111 adult patients with *T. tonsurans* TC confirmed by fungal culture or PCR, 95 patients received 250 mg/day of terbinafine for an average of 7.2 weeks. Of these, 83.2% (n = 79) of patients achieved clinical cure, while 16.8% (n = 16) were considered to have failed treatment [37]. In a sub-analysis among patients who failed treatment vs. those who were clinically cured, patients who failed treatment were more likely to also have tinea corporis (50% vs. 20.3%, OR 3.9, 95% CI 1.3–12.1, *p* < 0.02). Notably, the location and clinical subtype of TC infection were not associated with treatment failure or success [37]. In a retrospective study of 26 adult and pediatric patients with non-inflammatory *M. canis* TC confirmed by microscopy and culture, including four adult women aged 62–75 years, treated with terbinafine 250 mg/day, all adult patients achieved complete cure in 8 weeks without evidence of relapse, suggesting that a course of terbinafine for 8–12 weeks is an acceptable alternative for TC treatment in adults who have contraindications to griseofulvin [76].

In a retrospective study of 17 cases of *M. canis* (n = 8), *T. violaceum* (n = 4), *T. mentagrophytes* (n = 4), and *T. verrucosum* (n = 1) TC in women aged 17–76 years at a single center in Italy, treated with either griseofulvin 25 mg/kg/day or terbinafine 250 mg/day, and topical antifungal creams twice daily after shaving the hair once weekly, complete cure was achieved in 40–50 days with only a single case of relapse [77]. In a meta-analysis of seven studies including 2163 pediatric and adult subjects with TC diagnosis confirmed by microscopy and/or culture, pooled data analysis showed no difference in efficacy between terbinafine and griseofulvin (odds ratio 1.22, 95% CI 0.785–1.919, *p* = 0.37). Sub-group analysis for treatment of *Microsporum* species (n = 3 studies) indicated that griseofulvin was more efficacious compared to terbinafine (OR 2.45, 95% CI 1.52–3.94, *p* < 0.001), while sub-group analysis for treatment of *Trichophyton* species (n = 4 studies) indicated that terbinafine was more efficacious compared to griseofulvin (OR 1.616, 95% CI 1.274–2.051, *p* < 0.002) [78].

Itraconazole and fluconazole are off-label therapies for TC in the US. In a retrospective study of 58 cases of adult TC at five dermatology centers in Egypt, with 26 patients treated with 200 mg/day of itraconazole for 2-3 weeks (n = 6 treatment-naïve patients, n = 20 patients previously treated with griseofulvin or terbinafine), 80.8% achieved complete cure [31]. In a prospective study of 50 subjects, including 2 adults, with TC (*T. tonsurans* n = 41, *T. violaceum* n = 7, *T. soudanense* n = 1, and *T. rubrum* n = 1) treated with itraconazole pulse therapy (dose 200 mg per day for adults, each pulse 1 week in duration, with 2 weeks between pulse weeks 1 and 2, and 3 weeks between pulse weeks 2 and 3), complete cure was achieved in 81% of subjects (30 of 37 subjects with follow up information available). Most patients had moderate or severe disease (n = 16 and n = 7, respectively), and required two or three pulses (n = 14 and n = 13, respectively) of itraconazole. As such, the recommended dose of itraconazole for adults with TC is 200 mg/day for 2–3 weeks, delivered in either continuous or pulse doses [79]. Fluconazole as a treatment for TC in adults specifically has not been extensively studied for TC but may be dosed at 6 mg/kg/day, with maximum dose 400 mg/day, in line with pediatric recommendations [75,80].

Antifungal shampoo may be used to prevent TC development in adults living in households with children affected by TC. Although large randomized controlled trials comparing antifungal shampoo options have not been performed in adult patients, case reports suggest that ketoconazole shampoo in conjunction with oral antifungal treatment may be useful in treating TC in adult patients [81,82]. Studies in children have shown that selenium sulfide shampoo 1% and ciclopirox shampoo 1% are also acceptable adjunctive treatment options for TC infection [83]. Antifungal shampoos are not recommended as monotherapy in treatment of confirmed TC cases (Table 2).

Adults may be identified as asymptomatic carriers of TC infection. Asymptomatic carriers serve as reservoirs of infection and may be responsible for relapsed disease or treatment failure in their contacts and household members. In a cross-sectional prevalence study of 56 patients, a majority of whom were African American pediatric patients with mycologic confirmed TC and 114 contacts, including 50 adults, 18 contacts were identified as carriers at the initial visit, 6 of which were adult contacts (33%). Of the 44 households studied, 14 (32%) had at least 1 asymptomatic carrier identified [74]. When an asymptomatic carrier is identified, it is recommended that they be treated with appropriate therapy, depending on the causative species and extent of growth on culture. Although treatment guidelines for asymptomatic carriers are not widely agreed upon, treating patients with low spore count on culture with antifungal shampoo alone may be sufficient, while those with moderate to heavy growth on culture may require oral antifungals to prevent progression to clinical disease [84]. In households in which one individual is diagnosed with TC, it is recommended that all household members use an antifungal shampoo for 4–6 weeks to prevent the acquisition of TC infection [5,74]. Furthermore, combs, hairbrushes, sheets, towels, and other household items should be thoroughly sanitized to prevent re-infection (Table 2).

## 12. Prognosis and Sequelae of Untreated Disease

The prognosis for TC in adults is typically excellent when adequate treatment is administered. When hair loss occurs secondary to TC infection, patients typically experience gradual hair regrowth after they achieve complete cure [85]. In the case series of three adults with culture-confirmed TC by Vidimos et al., all patients (n = 3) experienced regrowth of scalp hair following treatment [73]. However, in some cases, delayed diagnosis or inadequate treatment can result in scarring alopecia [85]. In a case series of four adult patients with culture-confirmed TC by Buckley et al., one patient who presented with a pustular scalp eruption had delayed diagnosis, was subsequently treated with terbinafine 250 mg daily for one month, but experienced scarring alopecia as a sequela [85]. Patients with untreated, non-inflammatory TC are at higher risk for kerion development [46,52]. Patients with inflammatory TC, including kerion and favus, are at higher risk for permanent sequelae of disease, including scarring alopecia. It is recommended that immunosuppressed persons be followed closely to ensure adequate treatment and avoid sequelae of residual disease [20].

In cases of treatment failure, patients can be counseled on the importance of medication adherence. It is recommended that the household is evaluated, including those who may serve as asymptomatic carriers of TC. In addition, pets and fomites may be considered as carriers of infection. It is also important to ensure adequate treatment of any comorbid tinea infections of anatomic areas other than the hair and scalp to avoid reintroducing infection.

In a prospective, cross-sectional survey study including 164 children aged 6–16 years old with clinical diagnosis of TC, 79 (48.2%) of participants had a children’s dermatology life quality index (CDLQI) score ≥ 6, indicating psychological impact on quality of life, with 28 (35.4%) scoring in a moderate range (CDLQI 11–20) and 4 (5.1%) in a severe range (CDLQI 21–30). Although a similar study has not yet been conducted in adults, it is likely that adult patients with TC infection would experience similar psychological impact of disease. Future studies are needed to elucidate the extent of psychological impact of adults with TC infection [86].

## 13. Antifungal Resistance

Over the past decade, dermatophyte infections resistant to treatment with topical and oral antifungal agents have emerged. The main species that have been recognized as responsible for this recalcitrant type of infection include *Trichophyton indotineae* (*T. indotineae*) and *T. rubrum* [87,88]. Infections caused by *T. indotineae* primarily include tinea corporis, tinea cruris, and occasionally tinea faciei, and are recognized as reaching epidemic levels on the Indian subcontinent [87,88,89]. Infections with *T. indotineae* have been reported worldwide [90]. *T. indotineae* infections are often severe and widespread, covering a large body surface area compared to less extensive tinea infections that are easily cured with standard antifungal agents. *T. rubrum* is the leading cause of onychomycosis worldwide, for which terbinafine is generally effective [87]. However, there are increasing numbers of terbinafine-resistant *T. rubrum* onychomycosis cases reported worldwide [87].

TC infections resistant to antifungal therapy have not been reported as frequently as tinea corporis and cruris infections. *T. indotineae* and terbinafine-resistant *T. rubrum* have not been described as affecting the hair and scalp specifically. However, given the widespread availability of over-the-counter antifungal therapies in many countries and overzealous antifungal prescribing practices, and use of antifungal therapy by both physicians and patients, it is likely that treatment-resistant TC infections could develop [91]. In a 2019 cross-sectional study in Karachi, Pakistan, samples of hair and scalp were collected from 207 patients with clinically suspected TC, including 53 adolescents and 40 adults, and antifungal susceptibility testing for fluconazole and terbinafine was performed. Of the 61 samples that showed dermatophyte growth on culture, 12 samples were resistant to fluconazole (19.7%), while 7 samples (11.5%) were resistant to terbinafine. *T. mentagrophytes* and *T. violaceum* were the two species isolated that most often exhibited resistance [92]. Therefore, there is the potential for the spread of treatment-resistant TC infections in adults.

When physicians encounter TC infections that fail to improve with adequate therapy and proper adherence to therapy, resistance should be considered. Physicians can contact local health departments in their respective countries for instructions on how to proceed when resistant TC infections are suspected [90].

## 14. Conclusions

TC infections in adults may be primary or may be transmitted from children. Some adults who are diagnosed with TC infections are immunosuppressed, and therefore it is important to recognize the clinical features of TC in adults to promptly make the diagnosis and avoid treatment delays. While TC infections resistant to antifungal therapy are rarely reported to date, antifungal resistance is rising among superficial fungal infections in general, and antifungal stewardship is necessary to ensure that resistance to treatment does not worsen among dermatophytes that cause TC. Antifungal stewardship is also paramount in curbing the spread of treatment-resistant tinea corporis, cruris, and faciei infections, which can lead to autoinoculation and persistent TC infection among adults. Guidelines are needed to provide recommendations on preferred treatment course durations and medication dosages to achieve complete cure.

## Figures and Tables

**Table 1:** Antifungal therapy for adult tinea capitis *.

Drug Name	Dose	Frequency	Laboratory Monitoring
Griseofulvin ultramicrosize	10–15 mg/kg per day (maximum 750 mg per day)	6–12 weeks	Liver function panel and complete blood count if therapy extends ≥ 8 weeks
Terbinafine	250 mg per day	4–12 weeks depending on species 4–6 weeks for *Trichophyton* species 8–12 weeks for *Microsporum* species	Liver function panel before therapy begins and again if therapy extends ≥ 6 weeks CBC if therapy extends ≥ 6 weeks
Itraconazole	5 mg/kg per day (maximum 400 mg per day)	2–3 weeks, or consider pulse therapy	Liver function panel before therapy begins and again at 4 weeks
Fluconazole	6 mg/kg per day (maximum 400 mg per day)	3–6 weeks	No laboratory monitoring required

* Note, optimal treatment regimens for adult tinea capitis are not well-known. Standardized guidelines do not exist. CBC: complete blood count.

**Table 2:** Management of tinea capitis in addition to oral antifungal therapy.

Group	Interventions	Frequency and Duration
Individual diagnosed with TC	Antifungal shampoo (ketoconazole 2% shampoo, selenium sulfide shampoo 1% or 2.5%, or ciclopirox 1% shampoo)	Twice weekly until complete cure
	Disinfection of fomites (manual cleaning and chemical disinfection)	As needed
Asymptomatic Carriers	Antifungal shampoo (ketoconazole 2% shampoo, selenium sulfide shampoo 1% or 2.5%, or ciclopirox 1% shampoo) if spore count low on culture	Twice weekly until complete cure
	Addition of oral antifungals if spore count high on culture	Follow oral antifungal instructions
Household contacts of children with TC	Antifungal shampoo (ketoconazole 2% shampoo, selenium sulfide shampoo 1% or 2.5%, or ciclopirox 1% shampoo)	Twice weekly for 4–6 weeks

TC: Tinea Capitis.

## Data Availability

No new data were created or analyzed in this study. Data sharing is not applicable to this article.
